# Supplementary material for: Renormalized mechanics and stochastic thermodynamics of growing vesicles
Source: ArXiv. 2025 May 28:arXiv:2503.24120v2. Originally published 2025 Mar 31. Preprint. [Version 2] (PMC11998854)
Supplement: Supplement 1 [file NIHPP2503.24120v2-supplement-1.pdf]

# Supplemental Material for "Renormalized mechanics and stochastic thermodynamics of growing vesicles"

Jordan L. Shivers,[1,2] Michael Nguyen,[1,2] Aaron R. Dinner,[1,2] Petia M. Vlahovska,[3] and Suriyanarayanan Vaikuntanathan[1,2]

[1] *The James Franck Institute, University of Chicago, Chicago, Illinois USA*
[2] *Department of Chemistry, University of Chicago, Chicago, Illinois USA*
[3] *Engineering Sciences and Applied Mathematics, Northwestern University, Evanston, Illinois USA*

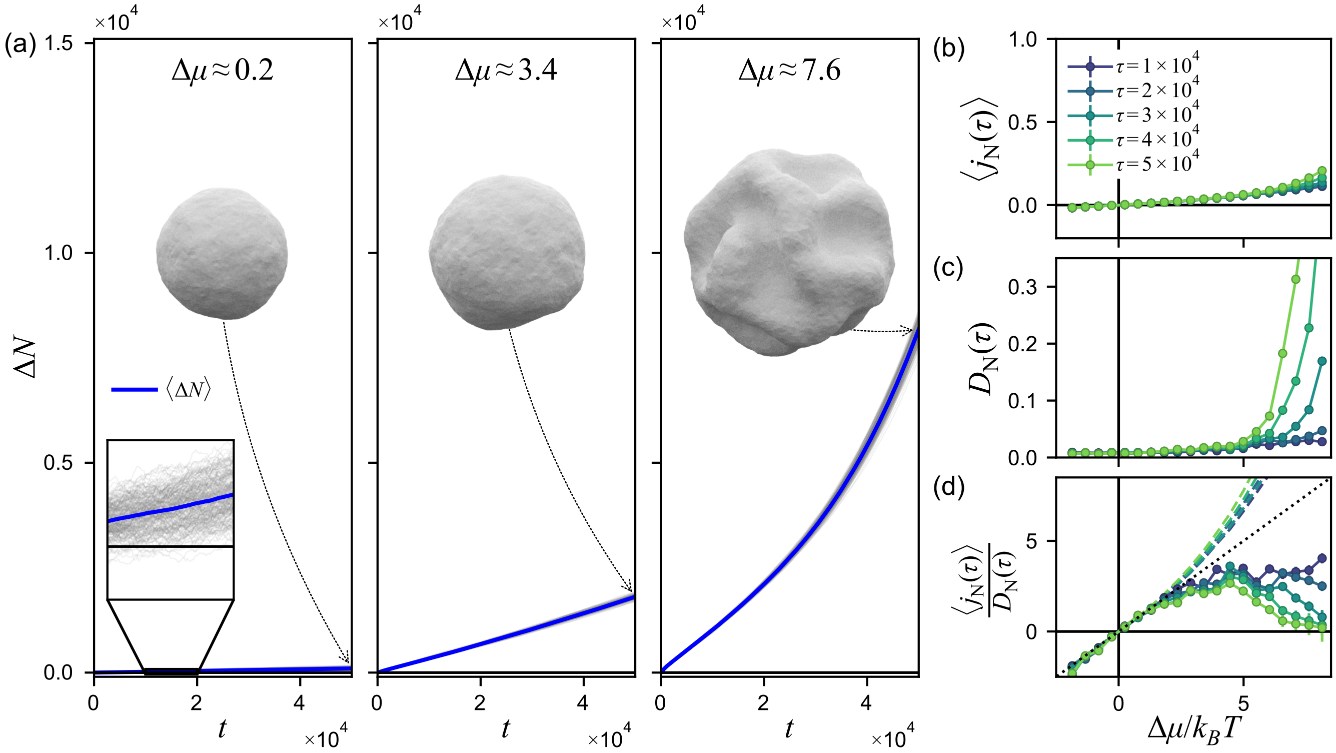


FIG. S1. **Growth dynamics with slower insertion/removal attempt rate of $p_{\mathrm{exchange}} = 0.01$.** (a) Ensembles of growth trajectories for varying values of excess chemical potential $\Delta\mu = \mu - \mu_{\mathrm{eq}}$, with the net influx of surface particles (vertices) ($\Delta N = N(t) - N(0)$) plotted as a function of the number of elapsed Monte Carlo sweeps $t$. Light gray lines correspond to individual trajectories and blue lines correspond to the ensemble average $\langle \Delta N(t) \rangle$. (b-c) The average current $\langle j_{\mathrm{N}}(\tau) \rangle = \langle \Delta N(\tau) \rangle / \tau$, the diffusivity $D_{\mathrm{N}}(\tau) = \mathrm{Var}(\Delta N(\tau))/(2\tau)$, and the ratio $\langle j_{\mathrm{N}}(\tau) \rangle / D_{\mathrm{N}}(\tau)$ are shown as a function of the excess chemical potential $\Delta\mu$ for varying cutoff times $\tau$. In (d), the dashed lines show the same ratio if the denominator is replaced with the equilibrium diffusivity $D_{\mathrm{N,eq}} = D(\Delta\mu = 0)$, and the diagonal dotted line corresponds to the linear response prediction of Eq. 11 in the main text. For these simulations, the particle reservoir exchange attempt rate is $p_{\mathrm{exchange}} = 0.01$, the imposed osmotic pressure difference is $\Delta p = 0$, and the number of samples is $n_{\mathrm{samples}} = 200$.

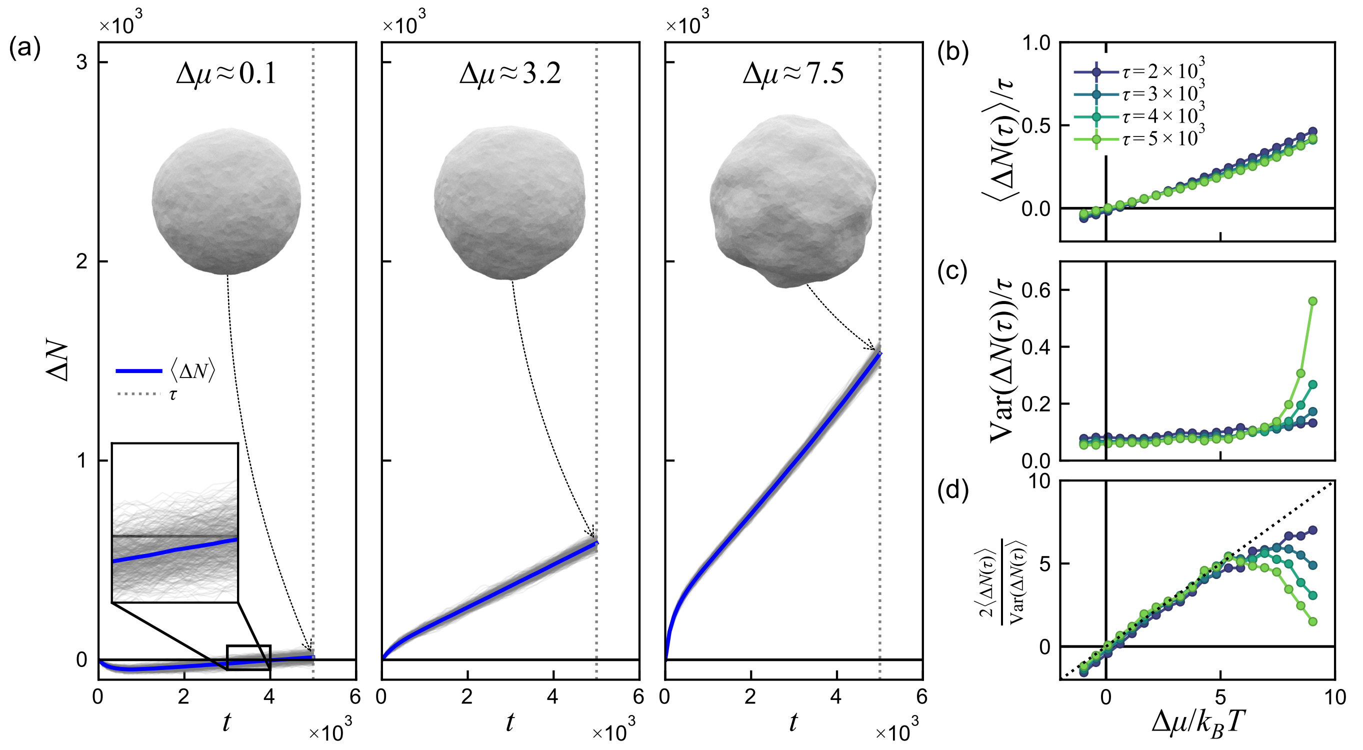


FIG. S2. **Growth dynamics with a positive pressure difference of $\Delta p = 0.4$.** (a) Ensembles of growth trajectories for varying values of excess chemical potential $\Delta\mu = \mu - \mu_{\mathrm{eq}}$, with the net influx of surface particles (vertices) ($\Delta N = N(t) - N(0)$) plotted as a function of the number of elapsed Monte Carlo sweeps $t$. Light gray lines correspond to individual trajectories and blue lines correspond to the ensemble average $\langle \Delta N(t) \rangle$. The vertical dashed gray curve indicates the cutoff time $\tau$. In (b-c), the average current $\langle j_{\mathrm{N}}(\tau) \rangle / \tau$, rate of increase in the variance $\mathrm{Var}(\Delta N(\tau))/\tau$, and the ratio $2\langle \Delta N(\tau) \rangle / \mathrm{Var}(\Delta N(\tau))$ are shown as a function of the excess chemical potential $\Delta\mu$ for varying cutoff times $\tau$. In (d), the diagonal dotted line is predicted by linear response. For these simulations, the particle reservoir exchange attempt rate is $p_{\mathrm{exchange}} = 1$, the imposed osmotic pressure difference is $\Delta p = 0.4$, and the number of samples is $n_{\mathrm{samples}} = 500$.

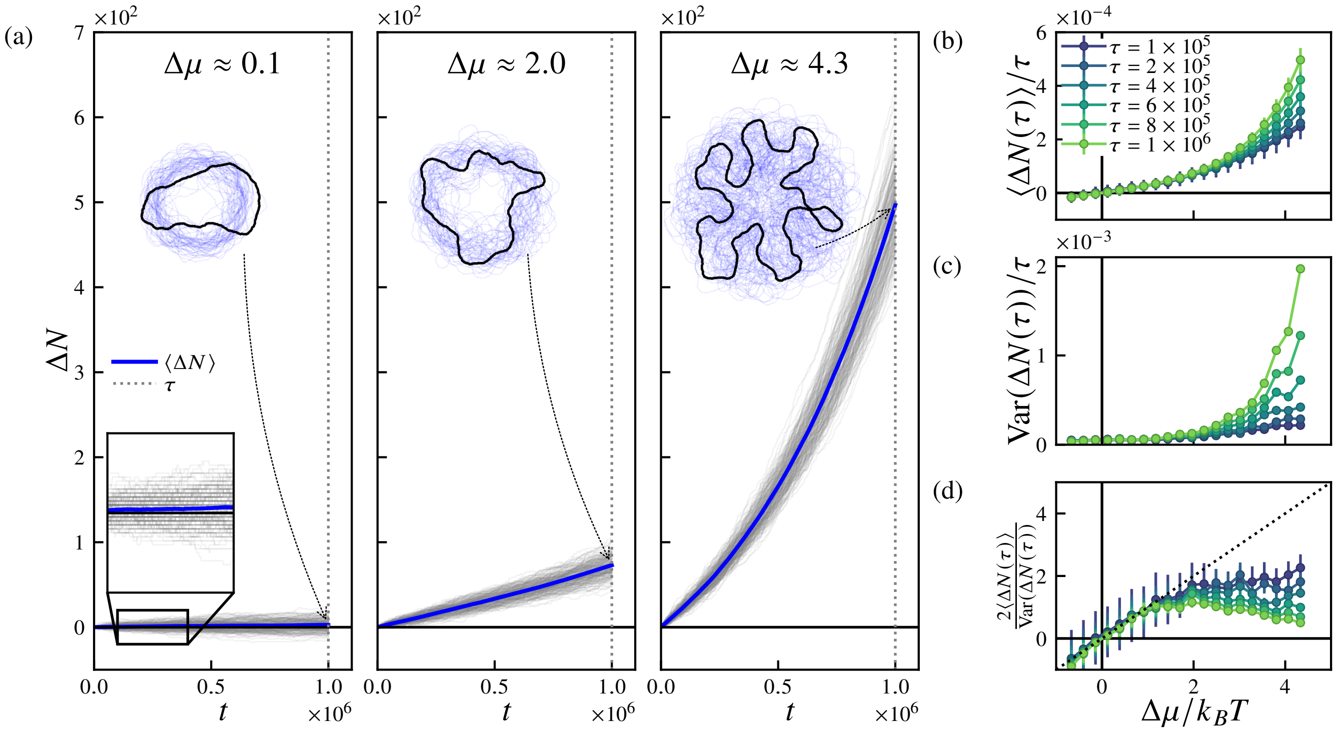


FIG. S3. **Growth dynamics for two-dimensional systems.** (a) Ensembles of growth trajectories for varying values of excess chemical potential $\Delta\mu = \mu - \mu_{\mathrm{eq}}$, with the net influx of surface particles (vertices) ($\Delta N = N(t) - N(0)$) plotted as a function of the number of elapsed Monte Carlo sweeps $t$. Light gray lines correspond to individual trajectories and blue lines correspond to the ensemble average $\langle \Delta N(t) \rangle$. Representative vesicle configurations are shown in the insets, illustrating the transition from circular to highly deformed morphologies with increasing $\Delta\mu$. The vertical dashed gray curve indicates the cutoff time $\tau$. In (b-c), the average current $\langle j_{\mathrm{N}}(\tau) \rangle / \tau$, rate of increase in the variance $\mathrm{Var}(\Delta N(\tau))/\tau$, and the ratio $2\langle \Delta N(\tau) \rangle / \mathrm{Var}(\Delta N(\tau))$ are shown as a function of the excess chemical potential $\Delta\mu$ for varying cutoff times $\tau$. In (d), the diagonal dotted line is predicted by linear response. For these simulations, the particle reservoir exchange attempt rate is $p_{\mathrm{exchange}} = 0.01$, the imposed osmotic pressure difference is $\Delta p = 0$, and the number of samples is $n_{\mathrm{samples}} = 200$.

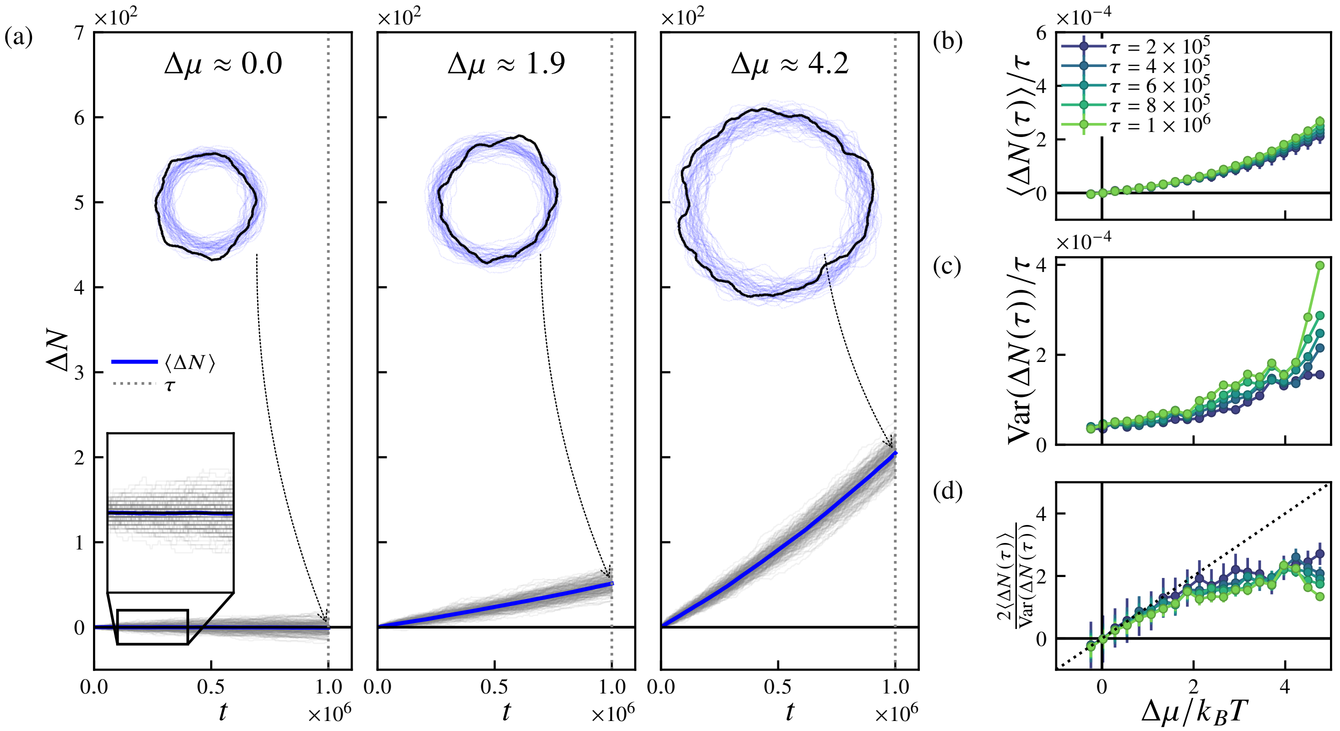


FIG. S4. **Growth dynamics for two-dimensional systems with a positive pressure difference of $\Delta p = 0.05$.** (a) Ensembles of growth trajectories for varying values of excess chemical potential $\Delta\mu = \mu - \mu_{\mathrm{eq}}$, with the net influx of surface particles (vertices) ($\Delta N = N(t) - N(0)$) plotted as a function of the number of elapsed Monte Carlo sweeps $t$. Light gray lines correspond to individual trajectories and blue lines correspond to the ensemble average $\langle \Delta N(t) \rangle$. Representative vesicle configurations are shown in the insets, illustrating that vesicles maintain approximately circular morphologies even at large $\Delta\mu$ due to the stabilizing effect of internal pressure. The vertical dashed gray curve indicates the cutoff time $\tau$. In (b-c), the average current $\langle j_{\mathrm{N}}(\tau) \rangle / \tau$, rate of increase in the variance $\mathrm{Var}(\Delta N(\tau))/\tau$, and the ratio $2\langle \Delta N(\tau) \rangle / \mathrm{Var}(\Delta N(\tau))$ are shown as a function of the excess chemical potential $\Delta\mu$ for varying cutoff times $\tau$. In (d), the diagonal dotted line is predicted by linear response. For these simulations, the particle reservoir exchange attempt rate is $p_{\mathrm{exchange}} = 0.01$, the imposed osmotic pressure difference is $\Delta p = 0.05$, and the number of samples is $n_{\mathrm{samples}} = 200$.

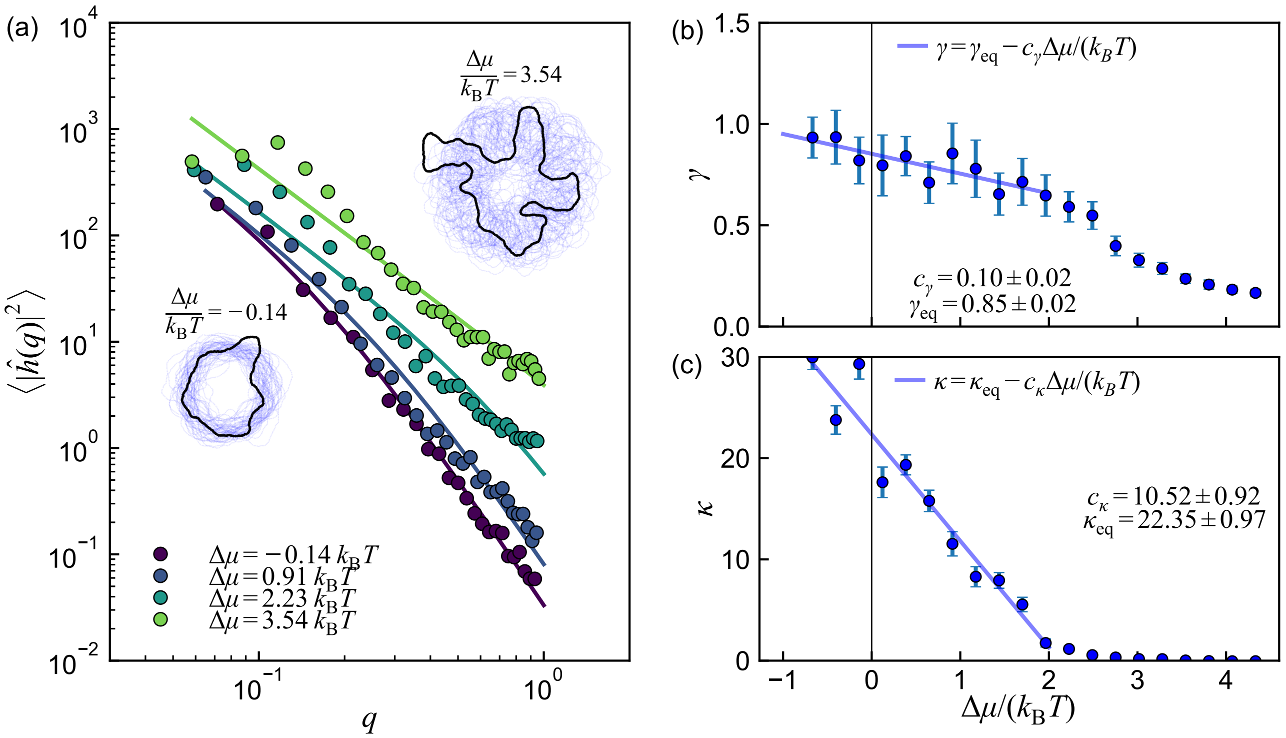


FIG. S5. **Power spectra and renormalized mechanical properties of growing 2D vesicles at varying excess chemical potential.** (a) Mean squared amplitude $\langle|h_q|^2\rangle$ of radial modes of degree $q$ for three different values of excess chemical potential $\Delta\mu$. Solid curves show fits to the theoretical model (Eq. 9 in the main text) with $\Delta\mu$-dependent renormalized tension $\gamma$ and bending rigidity $\kappa$. Representative vesicle configurations are shown in the insets. (b-c) Variation of the renormalized parameters with $\Delta\mu/k_B T$. The near-equilibrium regime ($\Delta\mu \approx 0$) shows approximately linear behavior characterized by slopes $\eta_\gamma$ and $\eta_\kappa$. Here, $p_{exchange} = 0.01$, $\Delta p = 0$, and $\tau = 10^6$ sweeps.

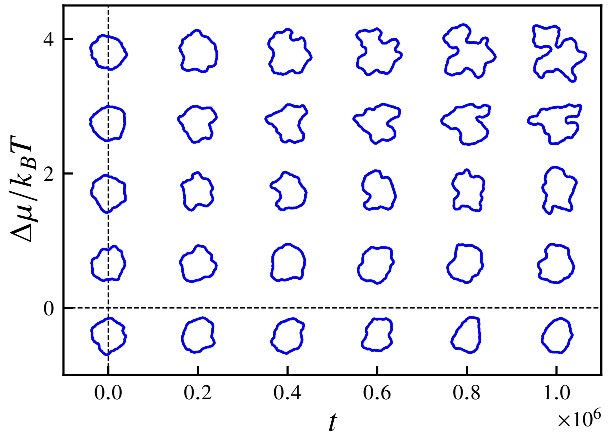


FIG. S6. **Configurational dynamics of 2D vesicles for varied imposed excess chemical potential.** Representative snapshots of vesicle configurations after number of sweeps $t$, for different values of the excess chemical potential $\Delta\mu/k_B T$ Images are centered on the corresponding values of $(t,\ \Delta\mu)$. For these simulations, the particle reservoir exchange attempt rate is $p_{exchange} = 0.01$, the imposed osmotic pressure difference is $\Delta p = 0$, and the average initial number of particles is $\langle N_0 \rangle = 200$.

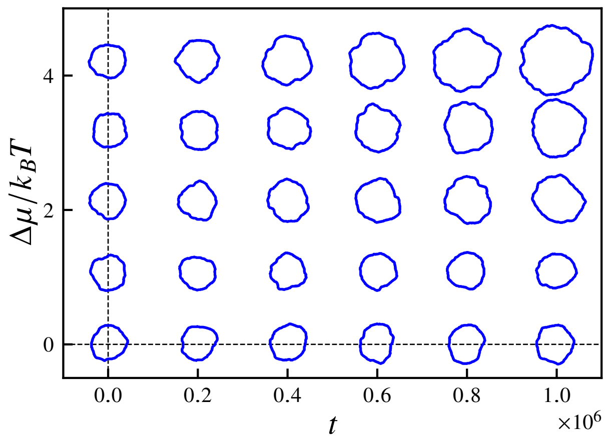


FIG. S7. **Configurational dynamics of pressurized 2D vesicles for varied imposed excess chemical potential.** Representative snapshots of vesicle configurations after number of sweeps $t$, for different values of the excess chemical potential $\Delta\mu/k_\mathrm{B}T$ Images are centered on the corresponding values of $(t,\ \Delta\mu)$. For these simulations, the particle reservoir exchange attempt rate is $p_\mathrm{exchange} = 0.01$, the imposed osmotic pressure difference is $\Delta p = 0.05$, and the average initial number of particles is $\langle N_0 \rangle = 200$.

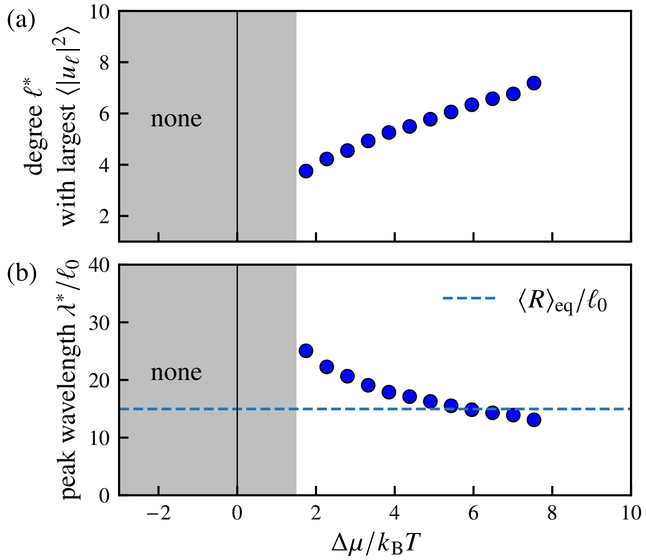


FIG. S8. **Dependence of the characteristic spherical harmonic degree and wavelength on nonequilibrium driving.** (a) Degree $\ell^*$ with largest mean squared amplitude $\langle |u_\ell|^2 \rangle$ as a function of the excess chemical potential $\Delta\mu/k_\mathrm{B}T$. In the region shaded gray, no dominant mode exists. (b) Peak wavelength $\lambda^*$ vs. $\Delta\mu/k_\mathrm{B}T$, showing the characteristic length scale of shape fluctuations.

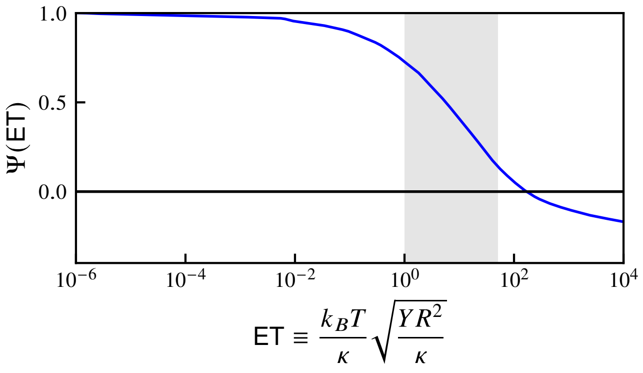


FIG. S9. **Renormalization group scaling function $\Psi(\mathrm{ET})$ for the buckling of externally pressurized elastic shells at finite temperature.** The scaling function $\Psi(\mathrm{ET})$, reproduced from Ref. 67 (main text), is plotted as a function of the elastothermal number ET. The range of elastothermal numbers seen in our simulations is highlighted in gray.

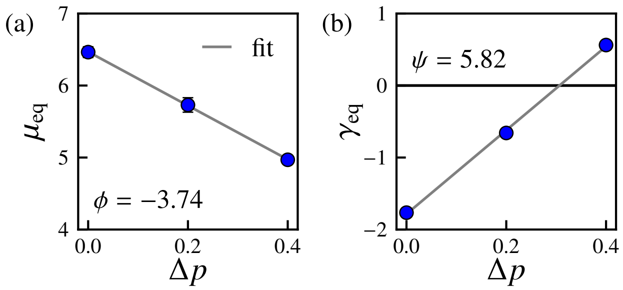


FIG. S10. **Dependence of the equilibrium chemical potential and effective tension on the imposed osmotic pressure difference.** The (a) equilibrium chemical potential $\mu_{\mathrm{eq}}$ and (b) equilibrium tension $\gamma_{\mathrm{eq}}$ are plotted as functions of $\Delta p$. Data points with error bars show measured values, while solid lines correspond to fits to the expressions $\mu_{\mathrm{eq}}\big|_{\Delta p} = \mu_{\mathrm{eq}}\big|_{\Delta p=0} + \phi\Delta p$ and $\gamma_{\mathrm{eq}}\big|_{\Delta p} = \gamma_{\mathrm{eq}}\big|_{\Delta p=0} + \psi\Delta p$, with slopes $\phi = -3.74$ (in units of $\ell_0^3$) and $\psi = 5.82$ (in units of $\ell_0$), respectively. For these data, $p_{\mathrm{exchange}} = 1$, $\tau = 5000$ sweeps, and the number of samples is $n_{\mathrm{samples}} = 500$.

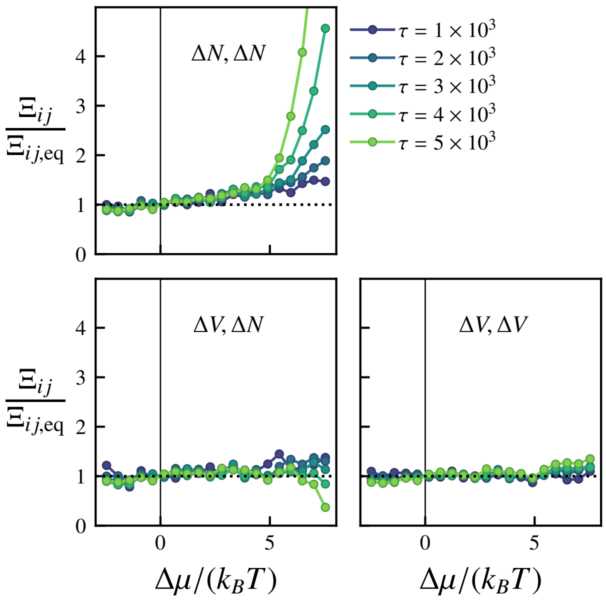


FIG. S11. **How do the components of the flux covariance matrix depend on cutoff time and distance to equilibrium?** The normalized component of $\Xi_{ij}/\Xi_{ij,\mathrm{eq}} = \mathrm{Cov}(J_i, J_j)/\mathrm{Cov}(J_i^{\mathrm{eq}}, J_j^{\mathrm{eq}})$ are plotted as functions of $\Delta\mu$ for varying cutoff times $\tau$. As $\boldsymbol{\Xi}$ is symmetric, only the lower triangular components are shown. For these data, $p_{\mathrm{exchange}} = 1$, $\Delta p = 0$, and the number of samples is $n_{\mathrm{samples}} = 500$.
